# Supplementary material for: Body Weight, BMI, Percent Fat and Associations with Mortality and Incident Mobility Limitation in Older Men
Source: Geriatrics (Basel). 2021 May 18;6(2):53. doi: 10.3390/geriatrics6020053 (PMC8162350; doi:10.3390/geriatrics6020053)
Supplement: Supplementary file 1 [file geriatrics-06-00053-s001.zip › geriatrics-1141546-supplementary.pdf]

Supplementary Table S1. Pearson correlation coefficients between weight, body mass index, ALM/ht^2 and percent body fat in older men.

	% fat	ALM/ht^2	Weight
BMI	0.68	0.74	0.87
Weight	0.60	0.67	
ALM/ht^2	0.11		
